# Supplementary material for: FtbZIP12 Positively Regulates Responses to Osmotic Stress in Tartary Buckwheat
Source: Int J Mol Sci. 2022 Oct 28;23(21):13072. doi: 10.3390/ijms232113072 (PMC9658761; doi:10.3390/ijms232113072)
Supplement: Supplementary file 1 [file ijms-23-13072-s001.zip › ijms-1984184-supplementary.pdf]

Supplementary File

FtbZIP12 positively regulates responses to osmotic stress in Tartary buckwheat

Wenfeng Weng [1], Xiang Lu [1], Meiliang Zhou [2], Anjing Gao [1], Xin Yao [1], Yong Tang [1], Weijiao Wu [1], Chao Ma [1], Qing Bai [1], Ruiqi Xiong [1] and Jingjun Ruan [1*]

[1] College of Agronomy, Guizhou University, Guiyang 550025, China.

[2] Institute of Crop Science, Chinese Academy of Agriculture Science, Beijing 100081, China.

*Correspondence: jjruan@gzu.edu.cn.

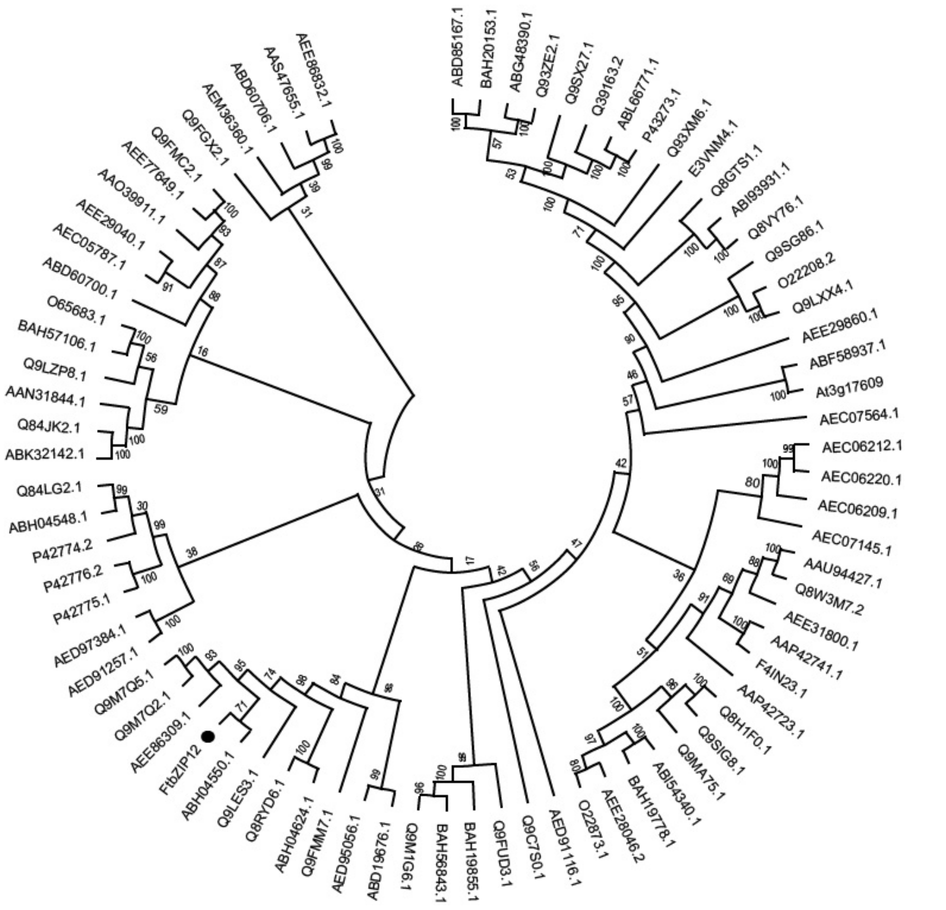

Figure S1. Phylogenetic tree analysis of *FtbZIP12* and the *Arabidopsis bZIP* family

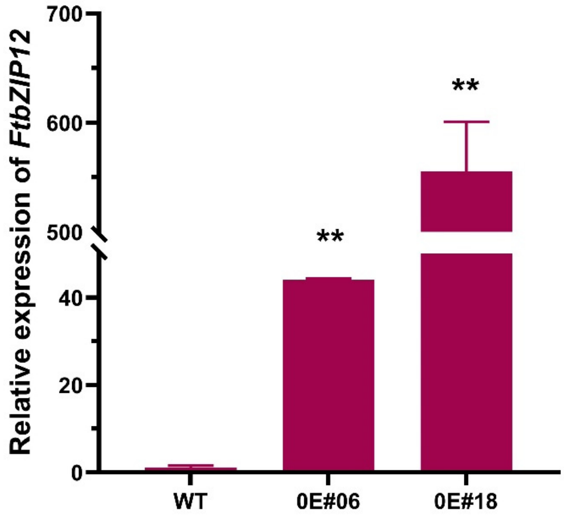

Figure S2. Identification of the relative expression of transgenic materials

Values are shown as means ± SD. ** $p < 0.01$ (Student's t-test).
